# Measuring the perceived quality of ophthalmology services in private organizations. A marketing perspective

**Published:** 2018

**Authors:** Iuliana Raluca Gheorghe, Consuela-Mădălina Gheorghe, Victor Lorin Purcărea

**Affiliations:** * “Carol Davila” University of Medicine and Pharmacy, Bucharest, Romania

**Keywords:** ophthalmology services, service quality, SERVQUAL scale

## Abstract

Nowadays, the competition registered on the Romanian markets regarding the activity of private ophthalmology organizations has raised their interest in developing consumer-oriented strategies. The key factor that assures a differentiation as well as a competitive advantage is the service quality from a marketing perspective.

Objectives: From a marketing perspective, service quality is measured as a perceived discrepancy between the consumers’ expectations and was actually performed in health care services. The most widely and validated measurement is the SERVQUAL scale. However, a variety of SERVQUAL scales have been applied in different health care environments without taking into consideration the specialty of the health care service. Thus, the objective of this paper was to measure the service quality in the Romanian ophthalmology private organizations using the SERVQUAL measurement, by identifying the SERVQUAL dimensions, which register the highest and the lowest gap scores.

Materials and methods: The instrument for data collection was the SERVQUAL self-administered questionnaire that consisted of 22 items measured on a 5-point Likert scale. The sample size encompassed 100 participants and the sampling technique was the snowball. The internal consistency, validity and the reliability of the SERVQUAL scale was determined by the Cronbach’s alpha coefficients and factor analysis. The SERVQUAL questionnaire focused on 5 dimensions (tangibles, reliability, assurance, empathy and responsiveness) and each dimension, in its turn, was characterized by different items.

Results: The mean age of the participants was 49.52 years, with a mean income of 3031 Romanian Currency and the mean period of wearing eyeglasses was 5 years (±2). Further, there were 47% females and 53% males. The overall internal consistency of the SERVQUAL scale, as well as the dimensions’ internal consistency were all above 0.7 and the factor analysis revealed that the items loaded properly on each dimension. Moreover, the gap scores of the SERVQUAL scale’s dimensions pinpointed that the highest gap score was registered by the Tangibles dimension and the lowest gap score was registered by the Reliability dimension.

Conclusions: Performing the ophthalmology service right the first time, contributes significantly to the improvement of the marketing effectiveness and the operating efficiency.

## 1. Introduction

Nowadays, the competition in private health care ophthalmology organizations has increased and, in order to survive, they have to deliver services that satisfy the consumer’s needs [**1**,**2**]. The key factor in differentiating services, which also assures a competitive advantage as well as consumer retention, positive word-of-mouth, increased profitability, financial performance and consumer satisfaction, is the service quality [**3**-**5**]. 

Studies in the health care field confirmed that high quality services are linked directly to increased market share, profits, and savings for an organization [**6**]. More exactly, since the 90s, the patients’ quality perceptions have accounted for 17-27% of the variation in a health care organization’s net revenue and asset returns [**7**]. 

By nature, healthcare is a credence service, patients being unable to assess the technical service quality accurately, therefore, functional quality is the primary judgmental feature. Thus, quality is a judgmental concept [**12**] and patients form their perceptions on the operational part of the quality [**13**].

In ophthalmology, service quality takes the same shape and has similar meaning to what was aforementioned about health services, in general, but it may register some differences related to the measurement scale.

During the 1980s, the service quality research increased and led to different empirical methods because service quality is almost impossible to measure [**14**]. However, the marketing experts determined the service quality based on the consumer perceptions. The most widely and validated scale in scientific literature is the SERVQUAL scale. Despite being a reliable instrument, many specialists consider it inappropriate, if used in health care services. 

Therefore, the objective of this paper was to measure the service quality in the Romanian ophthalmology private organizations using the SERVQUAL instrument. More specifically, we wanted to assess whether the SERVQUAL scale could be successfully applied in private ophthalmology services in Romania and determine the dimensions that register the highest gap scores.

**1. Literature review**

From a marketing perspective, perceived service quality is a concept that measures the discrepancy between the consumers’ expectations and their perceptions related to a health care service [**8**]. As such, expectations are reflected in the desires of the consumers that they believe a health care organization should provide. Once formed, expectations become important for consumers as they will help them make comparisons between what they anticipated and what they actually received [**9**]. On the other hand, perceptions refer to the consumer’s evaluation of the health care service provider, being considered, in fact, a combination between what is delivered and how it is delivered [**9**,**10**]. 

Still, like quality in most services, health quality is difficult to measure owing to the characteristics of services, namely intangibility, heterogeneity, and inseparability. Moreover, health quality perceptions rise according to the service size, complexity, specialization, and expertise within the health care organizations [**11**]. 

**1.1. Measures of service quality**

Considering the importance of service quality in health services, there is no surprise that many experts still spend a lot of their time understanding the underlying dimensions of the concept [**8**]. 

Many researchers concluded that from a consumer’s perspective service quality should be defined by two dimensions [**15**-**17**]. For example, Lehtinen and Lehtinen [**15**] determined the service quality in terms of corporate quality, interactive quality and physical quality whereas Gronroos [**16**] revealed the components of service quality as technical quality and functional quality, meaning what is delivered and how the service is delivered, respectively. In the same vein as Gronroos’ model approach [**16**], Berry [**17**] observed that service quality should encompass outcome quality and process quality. 

Following the research of other marketing specialists, Parasuraman et al. [**18**] elaborated the most widely used, validated and generally accepted service quality measurement in the services literature, the SERVQUAL scale [**19**]. The SERVQUAL measure is a multi-item instrument that consists of 5 dimensions, determined in their turn, by 22 paired items [**18**]. In fact, the SERVQUAL scale measures the expectation-perception gap of consumers [**20**]. Moreover, the gap score is the outcome of the difference between perception and expectation scores. Thus, a positive gap score suggests that the expectations of consumers have been exceeded whereas a negative gap score indicates failure. Further, gap scores are usually analyzed as aggregated scores giving an overview of each dimension and emphasize the strengths and weaknesses embedded in the actual service quality performance.

The five dimensions that define the SERVQUAL instrument are the following [**18**]:

- The Tangibles dimension that focuses on the physical facilities, the equipment used and the appearance of the personnel;

- The Reliability dimension suggests the ability of the service provider to perform the delivery of the service as accurately as promised;

- The Responsiveness dimension indicates an organization’s employees’ willingness to provide the consumers a prompt service;

- The Assurance dimension concentrates on an organization’s employees’ knowledge and courtesy as well as their ability to inspire trust and confidence to consumers;

- The Empathy dimension consists of the ability of the organization’s employees to provide caring and personalized attention to consumers. 

Despite the fact that SERVQUAL has been widely spread and has been considered a reliable instrument, many specialists criticized it both methodologically and conceptually. The most important criticism brought to light was that the five dimensions cannot be universally applied in all service industries and should be carefully implemented on each service market [**21**]. Moreover, the five dimensions should be restrained to two dimensions, namely the core services and augmented services [**22**] or to technical and functional dimensions [**16**]. 

**1.2. Health Service Quality**

As mentioned before, service quality remains a critical issue in most service industries and even more in health care services. Today, patient’s expectations changed as they became more informed and involved in the delivery of the service. As such, ensuring service quality is beneficial not only for the patients but also for the health care organizations. Investigating patients’ expectations would provide useful information for the health care provider who wishes to control and improve his service performance.

 In the health care sector, the traditional method to assess service quality is Donabedian’s structure-process-outcome model that includes the following dimensions [**23**]:

- Structure- includes the setting of the health care organizations;

- Process- suggests how health care is technically delivered;

- Outcome emphasizes the result of medical care on the health and welfare of the patient.

Donabedian’s model described quality as being more technical in nature rather than functional. In other words, it is not that commonly employed, as patients do not have the necessary medical knowledge to evaluate whether the health care service has been delivered properly. Moreover, there have been several attempts to assess the quality of a health care service based on two dimensions, but without any real success. For instance, even if technical quality has the highest priority, researchers resorted to measure it by proxy, helping patients make a difference between “curing” and “caring” services [**24**]. 

Despite all controversies related to the validity and reliability of the SERVQUAL scale, it proved to be efficiently applied in health care as well. Therefore, there have been shortened or extended versions of SERVQUAL with application in health care services in the scientific literature. For instance, Lim and Tang [**25**] extended the scale with 2 more dimensions, namely accessibility and affordability, Tucker and Adams [**26**] added caring and outcome while Johnston [**27**] shortened the initial SERVQUAL measurement, regrouping the items of the scale. Similarly, Tomes and Ng [**28**] integrated in the empathy dimension items that reflected understanding of the illness, relationship and mutual respect, dignity, physical environment and religious needs.

According to a research conducted by Purcărea et al. [**29**], who used the 22-item scale in measuring the health care service quality, it was concluded that the SERVQUAL scale might be successfully applied in this field as well. The mixed outcomes resulted from previous studies made us posited that the health service quality is far from being solved, more crucially if we were to take into consideration each medical specialty, such as ophthalmology. 

## 2. Materials and methods

After the elaboration of the SERVQUAL scale, many specialists applied it on health care services in the shape of 22-item format or modified, proving its usefulness in assessing the service quality as perceived by consumers in hospitals, clinics and other medical centers [**30**,**31**]. Further, most studies used the SERVQUAL self-administered questionnaire, selecting the sample participants from the lists with patients of the health care organizations, regardless of the medical specialty and their geographic location. In this research, we selected our sample respondents using the snowball technique but taking into consideration the following criteria:

- The respondents’ ages should have been more than 18 years;

- The respondents should have been wearing eyeglasses for more than 2 years;

- The respondents’ last consultation should have taken place in a private ophthalmology organization from Bucharest. 

The sample size was determined by using G*Power software and we concluded that a number of 150 participants should be enough to give us a clear overview of our researched objectives. From 150 participants, we validated 100 questionnaires, as many were not completely filled in or the respondents did not pay attention when filling in the questionnaire. 

The instrument for data collecting was the SERVQUAL self-administered questionnaire, consisting of two sections, as it follows:

- The first section collected information about the demographic profile of the respondents such as age, gender, marital status, income, reason for visiting an ophthalmology organization and the period of wearing eyeglasses.

- The second section encompassed the 22-paired questions that measured both expectations and perceptions on a 5-point Likert scale ranging from strongly agree (5) to strongly disagree (1).

The internal consistency, validity, and reliability of the SERVQUAL measurement was assessed by using the Cronbach’s alpha coefficient and the factor analysis was performed in SPSS version 20. 

In order to determine the SERVQUAL scale’s consistency and reliability, the threshold for the Cronbach’s alpha value was 0.7, which is the accepted limit [**32**], whereas for the factor analysis, we eliminated values of items lower than 0.4, which did not load properly on any latent factor, in our case being the SERVQUAL scale dimensions. Before performing the factor analysis, a preliminary statistical test was employed, namely the Kaiser-Meyer-Olkin (KMO) index accompanied by the Bartlett’s test of sphericity in order to examine the inter-correlated items. Moreover, the KMO test has to have values greater than 0.5 and the Bartlett’s test has to have a significant statistical level lower than 0.05. Consequently, the method used to uncover the latent variables, or in our case, the SERVQUAL scale’s dimensions, was the correlation matrix, with the Varimax rotation. 

## 3. Findings

**3.1 Demographic profile of the respondents**

The mean age of the participants was 49.52 years (±19.84), their mean income was 3031, 00 (± 1088) Romanian Currency and their mean period of wearing eyeglasses was 5 years (±2) (**Table 1**). Moreover, from 100 participants, 47% were females whereas 53% were males and went for a consultation to an ophthalmologist due to a routine check-up (22%), surgery (42%) and even asking for second opinions (36%) (**Table 2**). 

**Table 1:** The mean age, income and the period of wearing eyeglasses of the respondents

	Age of the respondent	Income of the respondent	Period of wearing eyeglasses
Mean	49,52	3031,61	5,00
Std. Deviation	19,847	1088,277	2,005
Minimum	18	711	2
Maximum	85	4922	8

**Table 2:** The distribution of the respondents’ genders according to the marital status and the reasons for seeing an ophthalmologist

Gender	Female	Male
Demographic variable	Frequency	Frequency
Marital status		
Not married	53.2%	45.3%
Married	29.8%	26.4%
Separated	17.0%	28.3%
Reasons for seeing an ophthalmologist		
Routine check-up	25.5%	18.9%
Surgery	36.2%	47.2%
Second opinion	38.3%	34%

**1.1 The SERVQUAL scale applied in private ophthalmology services**

**1.1.1 Internal consistency of the SERVQUAL scale **

The overall internal consistency of the SERVQUAL scale, as well as the dimensions’ internal consistency, was determined by the Cronbach’s alpha coefficients as illustrated in **Table 3**. As it can be observed, none of the scales had a Cronbach’s alpha coefficient lower than 0.7. 

**Table 3:** Cronbach’s alpha coefficients of the overall SERVQUAL scale, the expectation, and the perception scales as well as of every dimension included

Dimensions	No of items	Expectation	Perception
Tangibles	4	0,86	0,87
Reliability	5	0,90	0,89
Responsiveness	4	0,86	0,87
Assurance	4	0,89	0,87
Empathy	5	0,86	0,89
		0,78	0,73
		SERVQUAL scale: 0,82	

**3.1.1 Factor analysis**

According to the KMO index and the Bartlett’s test of sphericity, both the expectation scale and the perception scale had values higher than 0.80 with a significant level higher than 0.05., suggesting that the items included in the questionnaire were inter-correlated and the factor analysis was suitable to be performed. As such, the factor analysis for the expectation scale and the perception scale revealed that every item of the questionnaire loaded accordingly on each dimension (**Table 4** and **Table 5**). The dimensions of the expectation scale showed a 72.20% explanation of the variance whereas the dimensions in the perception scale explained 72.85% of the variance. 

**Table 4:** The Rotated Matrix of the Expectation Scale

	Reliability Dimension	Empathy Dimension	Component Assurance Dimension	Responsiveness Dimension	Tangibles Dimension
e_ta1					0.843
e_ta2					0.807
e_ta3					0.872
e_ta4					0.829
e_rel1	0.844				
e_rel2	0.854				
e_rel3	0.829				
e_rel4	0.829				
e_rel5	0.850				
e_resp1				0.813	
e_resp2				0.822	
e_resp3				0.846	
e_resp4				0.867	
e_ass1			0.879		
e_ass2			0.830		
e_ass3			0.851		
e_ass4			0.858		
e_emp1		0.818			
e_emp2		0.859			
e_emp3		0.844			
e_emp4		0.790			
e_emp5		0.723			

**Table 5:** The Rotated Matrix of the Perception Scale

	Empathy Dimension	Reliability Dimension	Component Assurance Dimension	Tangibles Dimension	Responsiveness Dimension
p_ta1				0.842	
p_ta2				0.869	
p_ta3				0.808	
p_ta4				0.854	
p_rel1		0.853			
p_rel2		0.828			
p_rel3		0.805			
p_rel4		0.810			
p_rel5		0.848			
p_resp1					0.853
p_resp2					0.857
p_resp3					0.841
p_resp4					0.822
p_ass1			0,862		
p_ass2			0,859		
p_ass3			0,844		
p_ass4			0,821		
p_emp1	0,807				
p_emp2	0,862				
p_emp3	0,849				
p_emp4	0,851				
p_emp5	0,848				

**1.1.1 The Gap analysis**

As mentioned earlier, the service quality is measured as the difference between the perception and the expectation of each dimension, namely the “gap” analysis. **Tables 6**-**10** indicate the gaps assessed for every dimension included in the SERVQUAL scale. 

**Table 6:** The Tangibles Dimension Gap Score

Items included in the Tangibles Dimension	Expectation Scale	Perception Scale	Gap Score (Expectation-Perception)
IT1	3,06	3,09	-0,03
IT2	3,08	2,95	0,13
IT3	3,18	2,97	0,21
IT4	3,13	3,09	0,04
Tangibles Dimension Gap Score:			0.35

**Table 7:** The Reliability Dimension Gap Score

Items included in the Reliability Dimension	Expectation Scale	Perception Scale	Gap Score (Expectation-Perception)
IT1	3,05	3	0,05
IT2	3	3,07	-0,07
IT3	2,99	3,03	-0,04
IT4	2,97	2,98	-0,01
IT5	3,07	3,08	-0,01
Reliability Dimension Gap Score:			-0,08

**Table 8:** The Responsiveness Dimension Gap Score

Items included in the Responsiveness Dimension	Expectation Scale	Perception Scale	Gap Score (Expectation-Perception)
IT1	2,83	2,87	-0,04
IT2	2,94	2,82	0,12
IT3	2,93	2,90	0,03
IT4	3,02	2,96	0,06
Responsiveness Dimension Gap Score:			0,17

**Table 9:** The Assurance Dimension Gap Score

Items included in the Assurance Dimension	Expectation Scale	Perception Scale	Gap Score (Expectation-Perception)
IT1	3,07	3,05	0,02
IT2	2,90	3,05	0,25
IT3	2,96	3,07	-0,11
IT4	3,06	3,14	-0,08
Assurance Dimension Gap Score:			0,08

**Table 10:** The Empathy Dimension Gap Score

Items included in the Empathy Dimension	Expectation Scale	Perception Scale	Gap Score (Expectation-Perception)
IT1	3,07	3,12	0,04
IT2	3,08	3,01	0,07
IT3	2,91	2,87	0,04
IT4	3,03	3,12	-0,09
IT5	3,04	2,99	0,05
Empathy Dimension Gap Score:			0,11

## 4. Discussion

The investigation of the quality in health care services has raised many controversies regarding their implementation and validation owing to their credence particularities. Based on different studies, the SERVQUAL scale has been the most widely used and employed in a vast array of fields. As such, the SERVQUAL scale is materialized in a gap measured between the expectations and perceptions of a consumer related to a health care service. 

From a marketing perspective, understanding the health care consumer in a competitive market, is highly essential as it may bring new insights in the health care industry overall. For instance, an improvement in the health care delivery of services can reduce the in-patient stays and lower mortality as well as deliver value to consumers. Therefore, identifying the dimensions’ gap scores may turn out to be useful from a strategic perspective, as they will indicate which components should be used as a competitive advantage. 

The SERVQUAL scale has been frequently applied in health care services without keeping in mind the specialty of the medical service but the type of health care organization. For example, Mangold and Babakus [**31**] measured the service quality in a US hospital, concluding that the assurance dimension has the lowest gap score whereas the empathy dimension has the highest gap score. Further, according to Lam [**30**], who applied the SERVQUAL scale in a hospital in Hong Kong, the highest gap score was registered by the empathy dimension and in a hospital located in Singapore, the dimension with the highest gap score was responsiveness [**25**]. In Romania, Popa et al. [**33**] determined the quality of health care services using the SERVQUAL scale in a hospital in Oradea and indicated that the empathy dimension registered the highest gap score whereas Purcarea et al. [**29**] confirmed that the tangibles dimension should be considered a competitive advantage. 

**Fig. 1:**
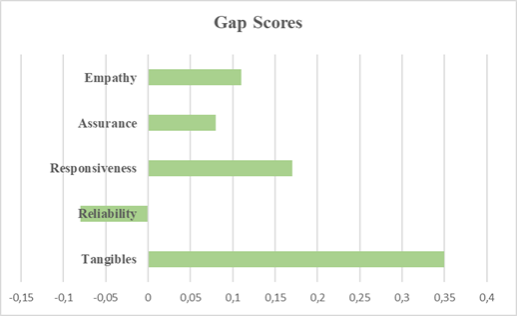
The SERVQUAL Scale’s Gap Score Dimensions

As mentioned earlier, none of the above studies took into consideration the specialty of the health care service. The objective of this paper was to measure the service quality in the Romanian ophthalmology private organizations using the SERVQUAL scale. More specifically, we wanted to identify whether the SERVQUAL scale could be successfully applied in ophthalmology services in Romania and determine the dimensions that register the highest gap scores. Thus, the highest gap score was registered by the Tangibles Dimension and the lowest gap score was registered by the Reliability Dimension (**Fig. 1**). 

**Fig. 2:**
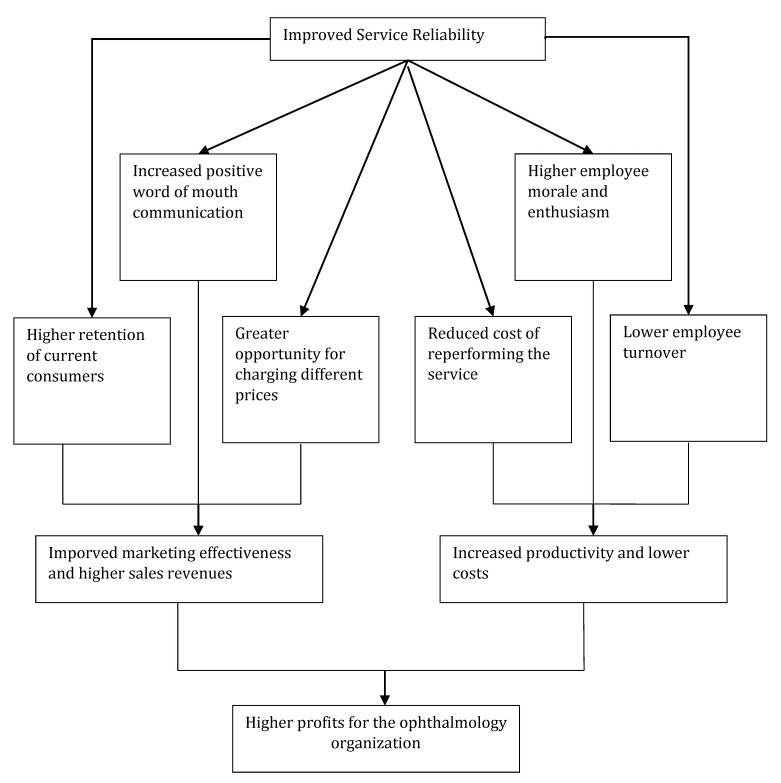
The benefits of performing a reliable ophthalmology service from a marketing perspective [**35**, p. 18]

According to some specialists, assuring reliability is paramount for every service industry and, in addition, is the essence of the service quality, which, in turn, is the core pillar for services marketing excellence [**34**]. Consequently, performing the ophthalmology service right the first time contributes significantly to the improvement of marketing effectiveness and the operating efficiency as well as to achieving higher current-consumer retention rates, increased word of mouth communication, and reduction in the need to reperform the service (**Fig. 2**).
